# Cervical Vertebral Body Implant Modification Accommodating Vertebral Artery Aneurysm Clips: A Case Report

**DOI:** 10.7759/cureus.105891

**Published:** 2026-03-26

**Authors:** Robert Rothrock, Vitaly Siomin, Rupesh Kotecha, Starlie C Belnap, Michael McDermott

**Affiliations:** 1 Miami Neuroscience Institute, Baptist Health South Florida, Miami, USA; 2 Miami Cancer Institute, Baptist Health South Florida, Miami, USA

**Keywords:** aneurysm clip model, cervical chordoma, implant surface modification, neurovascular surgery, spine surgery

## Abstract

Chordomas involving the cervical spine are best treated by surgical resection and post-operative radiotherapy. Excision of these tumors can be complicated by injury to surrounding soft tissues and neurovascular structures. We report a case of a cervical chordoma resection during which time the left vertebral artery was injured, requiring aneurysm clip sacrifice and the modification of the vertebral body implant to accommodate the applier end of the clips. The implant was modified with the removal of a small portion of one side to accommodate the proximal ends of aneurysm clips. The patient remained neurologically intact without cerebrovascular insult. Modification of the implant did not affect the structural integrity of the corpectomy implant, the planning of radiation therapy, or the final clinical result. At 2.5 years from surgery, the patient remains clinically well with no evidence of recurrence and no structural failure of the implant.

## Introduction

Chordomas are malignant primary bone tumors that can occur along the cranial and spinal axis but are most commonly seen in the clivus and sacrum. Cervical spine chordomas account for 10% of all chordomas and approximately 30% of all spine chordomas [1]. The incidence of chordomas in the population is 1/100,000, accounting for about 4% of malignant bone tumors, so they are "orphan tumors" in the spectrum of clinical malignancy [1]. Prognosis in patients with chordomas is usually poor with a five-year survival rate between 50% and 68% and a 10-year survival rate between 28% and 40% in the reported series [1,2]. Treatment of these tumors in the cervical spine requires maximal safe excision, reconstruction of bony elements to preserve stability and motion of the sub-axial spine, and post-operative radiotherapy to treat microinvasive disease into surrounding tissues [3]. Pre-operative planning when the tumor extends beyond the vertebral body can include computed tomography (CT) and magnetic resonance imaging (MRI), as well as angiography, but rarely embolization. Knowledge of the status of the vertebral arteries, and which artery is dominant, is important if the surgeon is considering elective sacrifice of the artery pre-operatively or if there is an intra-operative injury that is not repairable.

We report a case of a cervical spine chordoma operated on with plans for complete resection and reconstruction, where the left vertebral artery (VA) was injured and could not be repaired. Proximal and distal occlusion with permanent aneurysm clips required the modification of the polyetheretherketone (PEEK) implant in order to accommodate the proximal end of the aneurysm clips so they were not moved during the placement and fixation of the vertebral body implant. After the placement of aneurysm clips, the vertebral body implant would not fit into the vertebral body defect without significant rotation of the clips. The surgeons felt that the rotation of the clips might dislodge them from the proximal and distal ends of the transected VA and accordingly modified the implant by creating a cavity in one wall to accommodate the clip ends. Temporary suturing of the aneurysm clip loupes to the surrounding paravertebral muscles allowed the clips to be moved out of the way during positioning of the vertebral body implant, and once the implant was placed, the sutures were cut, and the clips rotated back into the cavity sculpted into the implant for this exact purpose.

## Case presentation

**Figure 1:**
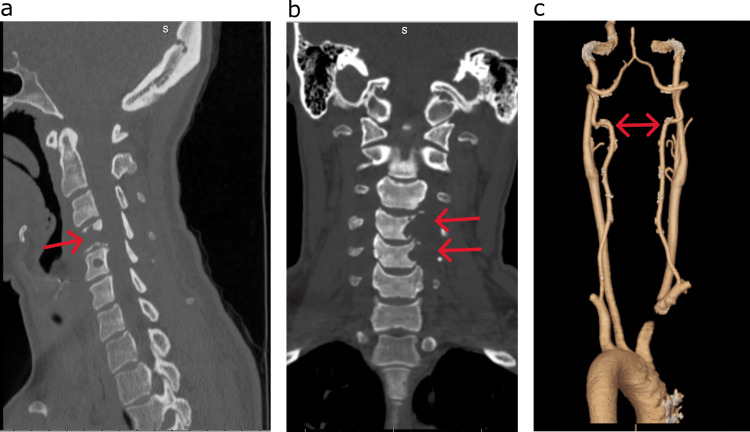
Pre-operative CT scans (a) Sagittal view demonstrating the left-sided C4-C5 erosive lesion (red arrow). (b) Coronal view demonstrating the left-sided C4-C5 erosive lesion (red arrows). (c) CT angiogram demonstrating the co-dominant vertebral arteries (red arrows). CT: computed tomography

The patient at the time of presentation was a 40-year-old woman who had progressive left-sided neck pain, shoulder pain, and left upper extremity radiculopathy in the C5 distribution, initially thought to have an intervertebral disc herniation. She however began to note weakness in the left hand. On clinical examination, the patient was noted to have 4+ out of 5 strength in the left-hand grip versus the right upper extremity. For this reason, she presented to the emergency department, where a CT scan of the cervical spine revealed an erosive lesion on the left-sided vertebral body of C4 and C5 (Figure 1). 

**Figure 2:**
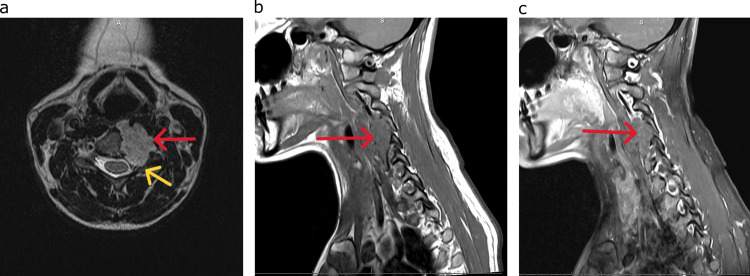
Pre-operative MRI scans (a) T2 axial view of the hyperintense lesion eroding the left C4 and C5 vertebral bodies (red arrow) with the compression of the left C4, C5 (yellow arrow), and C6 nerve roots. (b) T1 sagittal view of the pre-contrast image of the left-sided C4-C5 lesion (red arrow). (c) Sagittal post-contrast T1 image with mild enhancement of the left-sided C4-C5 lesion (red arrow). MRI: magnetic resonance imaging

An MRI study was completed demonstrating a T2 hyperintense lesion with contrast enhancement eroding the C4 and C5 left-sided hemi-vertebral bodies and compressing the left-sided C4, C5, and C6 nerve roots. Additionally, the lesion was noted to displace the left-sided VA anterolaterally (Figure 2). Due to the involvement of the tumoral lesion of the left-sided VA, a CT angiogram of the head and neck was performed (Figure 1c). 

This revealed co-dominant vertebral arteries and a full circle of Willis with bilateral posterior communicating arteries. The chief differential diagnosis given the radiographic appearance was chordoma, with consideration also given to schwannoma, as well as other primary spinal tumors such as chondrosarcoma or giant cell tumor. The patient also underwent positron emission tomography (PET)/CT study which did not demonstrate any other lesions; therefore, this was not felt likely to be metastatic disease. Biopsy was not favored due to the potential high risk of injury to the VA based on discussion with interventional radiology. 

Given the patient's young age, anticipated diagnosis of chordoma, and desire for maximal resection based on anticipated intra-operative pathology, the patient was offered anterior-posterior surgical intervention for the resection of this more aggressive-appearing malignancy. This was offered in the form of C4-C5 anterior cervical corpectomy, with anterior cervical instrumentation and C3-C6 fusion. This would then be followed by a stage II surgical intervention during the same surgical procedure in the form of a C2-C7 instrumented fusion. Pre-operative left-sided formal catheter diagnostic angiography was not performed due to adequate imaging with a CT angiogram. Additionally, ideally, the goal during surgical resection was the preservation of the left-sided VA; therefore, pre-operative embolization and sacrifice of the left-sided VA were not performed.

**Figure 3:**
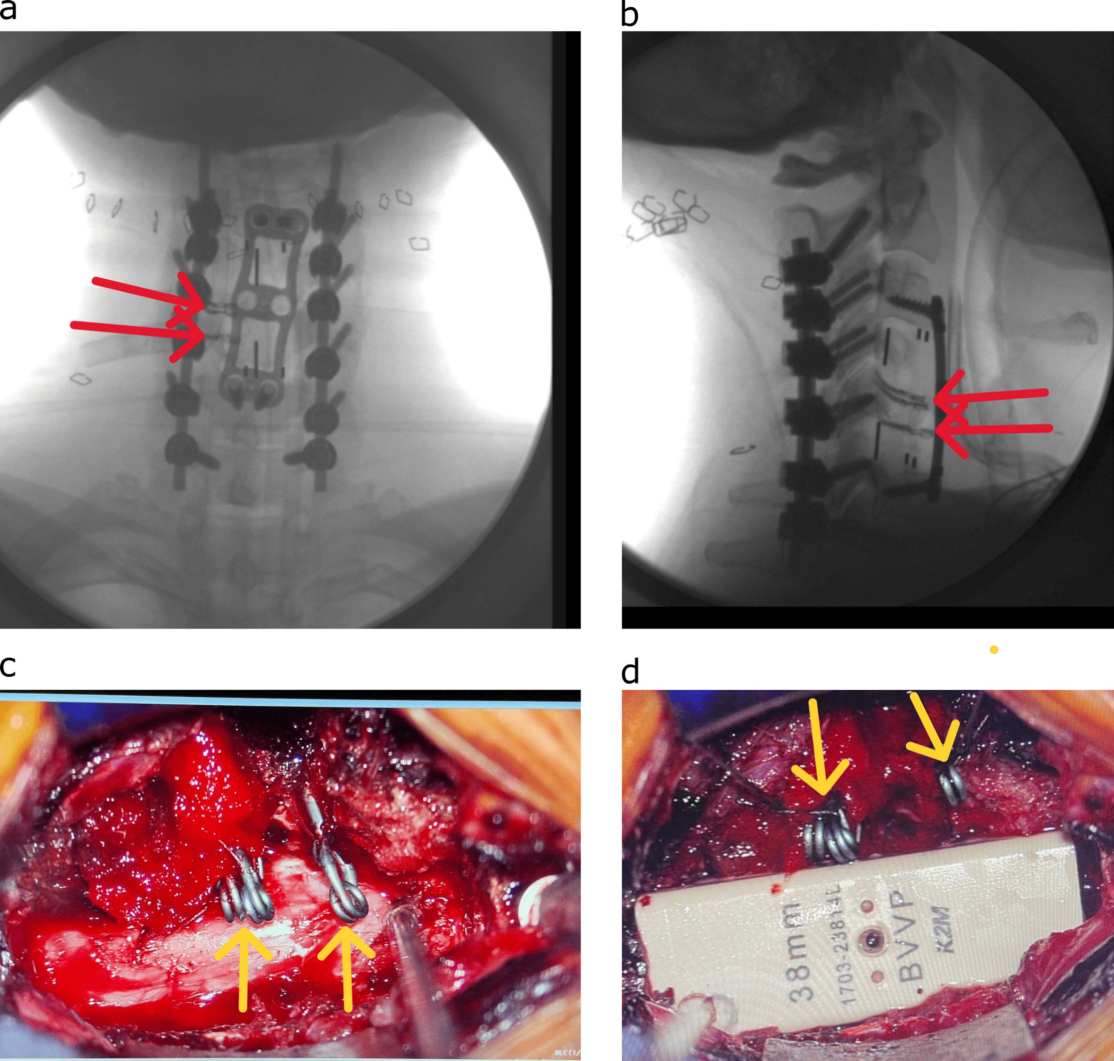
Intra-operative images (a) Intra-operative X-ray (anterior-posterior view) demonstrating the anterior-posterior and lateral images of the operative construct. Note the presence of the three aneurysm clips (red arrows). (b) Intra-operative X-ray (lateral view) demonstrating the anterior-posterior and lateral images of the operative construct. (c) The aneurysm clips underlying the surgically modified interbody cage (yellow arrows). (d) Interbody cage. Note the presence of the three aneurysm clips (yellow arrows).

Following the removal of the C4 and C5 vertebral bodies, attention was turned towards lateral tumor resection on the left side. During this anterior tumor resection, the left VA was injured on the anterior and posterior walls of the vessel with the ultrasonic aspirator. Inspection by our cerebrovascular surgeon determined that direct repair was not possible, and permanent aneurysm clips were applied proximal and distal to the arterial injury, beyond the inferior and superior ends of the tumor. After complete tumor excision, which included the removal of the C4 and C5 vertebral bodies, it became obvious that the proximal ends of the clips would interfere with the placement of the PEEK vertebral body corpectomy implant. Therefore, an iterative process of sculpting the posterior and left side of the implant followed by trial positioning was done until it appeared that the cavity created in the implant would accommodate the proximal ends of the clips. Prior to the final positioning of the implant, a 4-0 suture was placed through the loupes of the proximal and distal clips, and then the clip loupes were sutured laterally to the paravertebral muscles to rotate them out of the way of the implant. Prior to final plate fixation, the sutures were cut, and the clips rotated back into the cavity while hemostasis was maintained (Figure 3).

**Figure 4:**
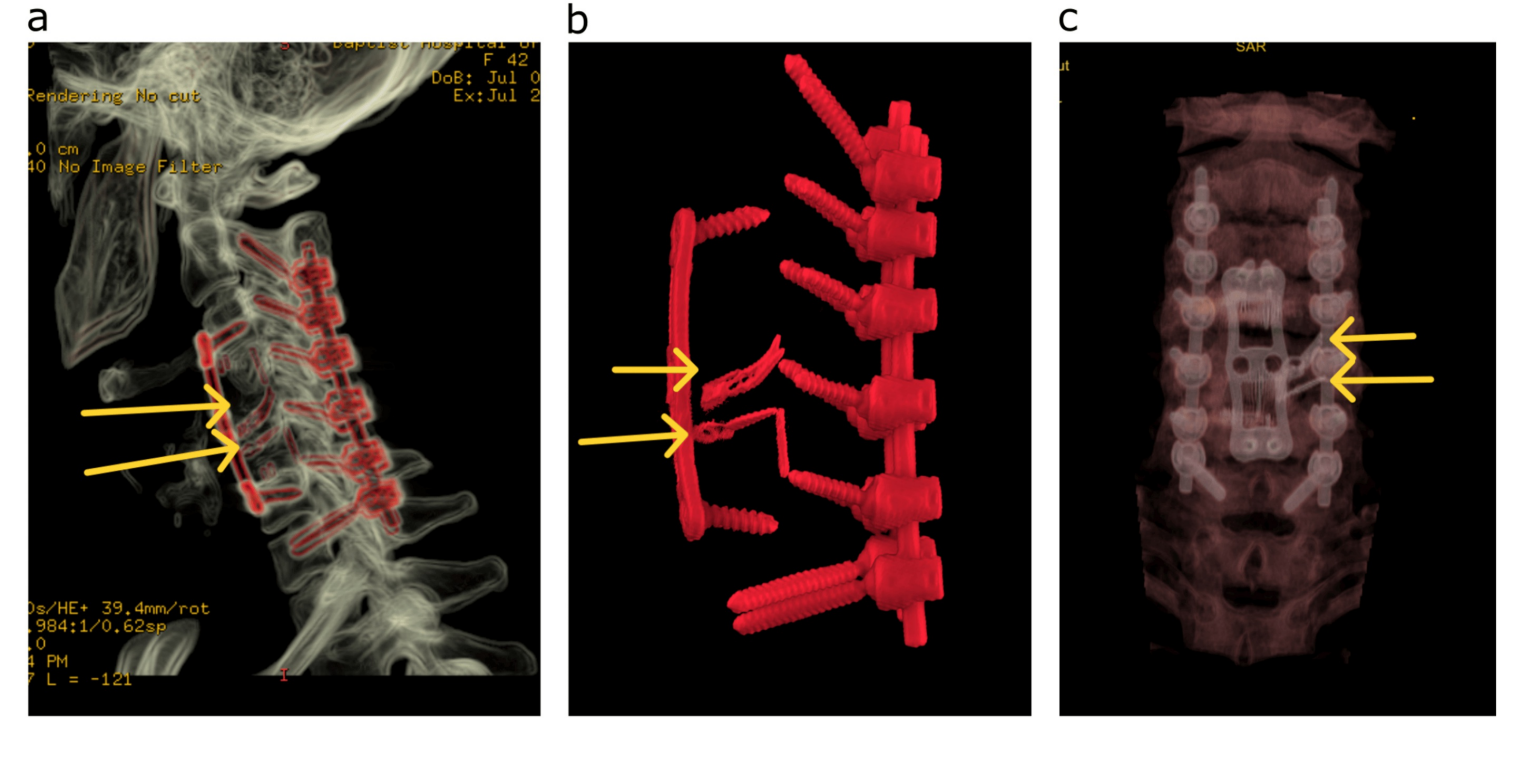
Post-operative CT reconstructions (a) Post-operative CT scan reconstruction (yellow arrows with aneurysm clips). (b) Subtracted reconstruction demonstrating the intra-operative modification of the PEEK interbody cage and accommodating the aneurysm clips (yellow arrows) on the sacrificed left vertebral artery. (c) Coronal reconstruction showing the construct (yellow arrows to aneurysm clips). CT: computed tomography; PEEK: polyetheretherketone

**Figure 5:**
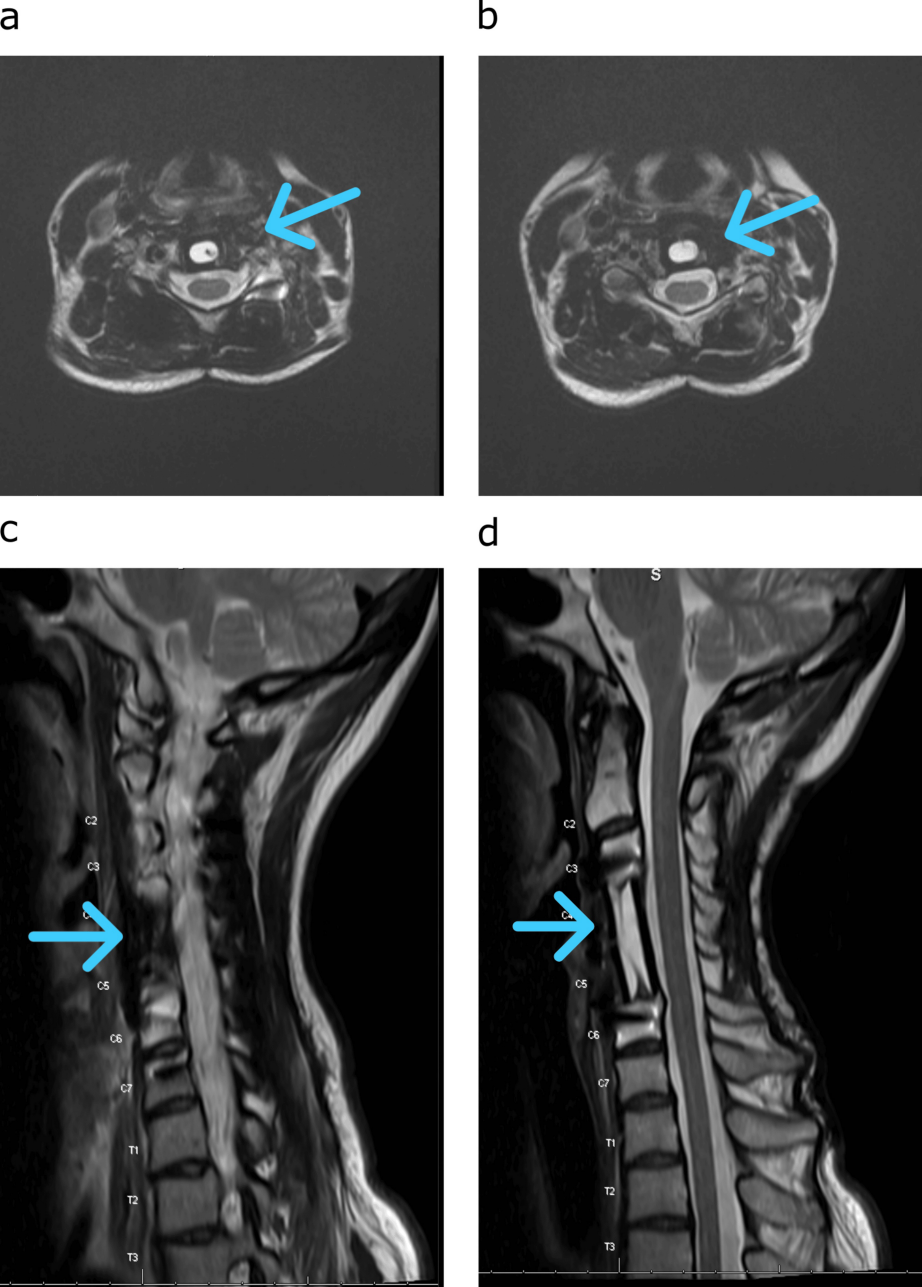
Post-procedure follow-up MRI scans Delayed 27-month follow-up MRI studies demonstrating continued resection and local control of the cervical chordoma. (a and b) T2 contrast-enhanced axial images at two different slices (blue arrows with the absence of tumor). (c and d) T2 contrast-enhanced sagittal images at two different slices (blue arrows with the absence of tumor). MRI: magnetic resonance imaging

Following surgery, the patient remained neurologically intact, and hybrid post-operative proton beam radiotherapy and stereotactic body radiation therapy were administered (Figure 4). She received a total dose of 73.8 Gy in 41 fractions. During the last follow-up at 27 months, the patient remains free of recurrence (Figure 5) without any new neurological deficit.

## Discussion

Cervical spine chordoma is an overall rare clinical entity and further complicated by the surrounding local regional anatomy, including the spinal cord, radicular nerve roots, proximal brachial plexus, vertebral arteries, and carotid arteries, amidst many other nervous, vascular, and lymphatic structures [4]. Effective surgical treatment and management includes ideally gross total resection versus aggressive intralesional resection and post-operative adjuvant radiation treatment and therapy [5]. The most common presenting symptoms of cervical chordoma include radicular pain and/or motor deficit [5]. By the time of presentation, most lesions are sizable with the median tumoral diameter at presentation reported as 3.9 cm [5]. Additionally, involvement and/or extension to the VA canal has been reported at up to 70% incidence [4]. Therefore, involvement and/or concern for VA injury is a common concern in the setting of surgical intervention for cervical spine chordoma [6,7]. In the modern era, the five-year overall survival rate is favorable at approximately 82%; however, there is still substantial 10-year mortality with the 10-year overall survival rate at approximately 53% [4]. Most if not all interventions tend to include instrumentation given the high degree of mobility of the sub-axial cervical spine. Surgical complications include hardware failure or fracture, spinal cord injury, cerebrospinal fluid leak, brainstem infarction secondary to VA injury, meningitis secondary to cerebrospinal fluid leak, and vocal cord paralysis versus dysphagia and dysphonia with the anterior approach [4]. Although less common, chordoma also poses a risk of distant metastasis, with a reported incidence of up to 9% of cases with metachronous metastatic disease formation [7]. The incidence of unintended VA injury in the setting of cervical spine chordoma is reported to be up to 5% [7].

The authors of this manuscript did not find any studies that would specifically address the incidence of VA injury in patients with cervical spine chordoma, which is not surprising, given the rarity of this condition. It obviously depends on the size and extent of the tumor, the cervical spine level, and the surgical goals (i.e., aggressive en bloc resection vs palliative debulking). There is a growing body of literature reporting on the incidence of the VA injury in patients undergoing surgical interventions to treat other, more common conditions, such as trauma and degenerative processes. Based on this literature, the overall incidence of iatrogenic VA injury is fortunately low (0-1.4%) [6-8]. The most common immediate causes of VA injury are drilling in the vicinity of the artery (20.6-61%) or use of other surgical tools and implantation of devices (16-31.4%) [7]. Arterial damage during soft tissue manipulation, detachment of the ossified posterior longitudinal ligament, and aggressive utilization of diathermy account for the rest of the cases [8]. In certain cases, the VA injury may not become apparent until some time after surgery. Wu et al. described a case of VA pseudoaneurysm diagnosed three weeks after the subtotal resection of the chordoma at the cranio-cervical junction [9]. The pseudoaneurysm was treated with endovascular coiling [9].

In the reported case in this manuscript, during resection of the chordoma, the ultrasonic aspirator perforated the front and back wall of the VA creating a defect that could not be repaired by an end-to-end anastomosis. The portion of the one wall of the implant resected was less than 20% of the surface area on that side and was not felt to pose a problem with the structural integrity of the implant. At 2.5 years of follow-up, the patient is clinically asymptomatic without evidence of recurrent tumor and no evidence of structural failure of the implant.

The most commonly injured segments of the VA are at the anterior portion of C7, the lateral segments of C3-C7, and the posterior segments of C1 and C2 vertebrae [10]. VA dominance is another factor to consider in surgical planning and management. In 50% of the human population, the dominant VA is on the left side, whereas 25% of the subjects have an equal diameter of the vessels [10]. When VA dominance is present, the probability of the ischemic changes in the posterior circulation areas is significantly higher in the distribution of the posterior inferior cerebellar artery (11.5% vs 1.5%) in patients without VA dominance and basilar artery distribution (20.5% vs 7.4%) [10].

In the rare and unfortunate event of the intra-operative VA rupture, the surgeon's focus quickly switches from the solemn beauty of the statistics and academic pearls to preserving the patient's life. Obtaining control of "torrential" bleeding from a large vessel frequently encased by a bulk of tumor tissue distorting the normal anatomy may become a very challenging task. It always helps to have an experienced colleague scrubbed alongside with large-bore suction. Mechanical pressure and the use of various hemostatic agents can help to decrease the bleeding rate and dry the surgical field while trying to remove more tumor to free expose the arterial tear and, ideally, secure proximal and distal control. The latter may not be easy in cases of a narrow and/or very deep exposure. Once the bleeding has stopped, having a cerebrovascular surgeon to assess the ruptured vessel may be very helpful, depending on the size and shape of the violation. The decision is made on whether to repair the vessel primarily with the use of microsurgical suturing techniques, apply a non-occlusive clip, or sacrifice it. In the reported case, the vessel was deemed unrepairable, and the decision was made to sacrifice it, with the pre-operative verification of adequate collateral blood supply. Aneurysm clips have an advantage over other types of clips as they can be precisely directed and readjusted/removed, if needed, in a deep and tight space. Once sacrificed, the information on the VA dominance may become relevant in prognostication. Endovascular repair of the injured VA, though technically possible, with the stent (+/- coiling of pseudoaneurysm) requires the subsequent administration of antiplatelet agents, which can be problematic in patients after extensive surgical manipulation and with large wounds.

Several other recommendations with regard to the management of the VA can be found in the literature. Barrenechea et al. suggested to preserve the VA whenever possible, as in case of local recurrence on the contralateral side, the surgical management may be considerably limited due to the presence of only one functional VA [3]. Hsieh et al. discussed the importance of pre-operative assessment of the intracranial blood supply with catheter angiography and recommended the performance of the balloon test occlusion (BTO) to evaluate the feasibility of VA sacrifice [11]. Pre-operative angiography was not performed in the reported case. In retrospect, it could potentially be beneficial for reducing the level of anxiety when the arterial injury occurred. On the other hand, the vessel sacrifice was not planned, and the authors find it hard to make a strong general recommendation to perform pre-operative angiography with or without BTO in all chordoma patients, as these endovascular interventions are not completely risk-free and benefits may not necessarily outweigh the risks, unless VA injury/sacrifice is felt to be likely and/or beneficial from the extent of resection standpoint. It should be noted that VA preservation was the primary goal in this particular case. Hence, given the known collateral blood supply and lower risk, BTO was not performed in this clinical circumstance.

Radiation therapy for patients with spine chordomas is especially challenging given the high doses of radiation therapy required and the nearby sensitive central nervous system (CNS) organs at risk (OARs) as well as the OARs in the surrounding head and neck region. Conventionally fractionated low-dose photon radiotherapy (<70 Gy full dose) has been associated with poor disease control rates. A study from the National Cancer Database on 282 chordoma patients treated to a median dose of 58 Gy demonstrated no improvement in overall survival with the addition of adjuvant radiotherapy [12]. Moreover, a meta-analysis of 193 patients at four high-volume centers treated to a median dose of 61.8 Gy demonstrated no improvement in local control with adjuvant radiotherapy; however, a dose ≥70 Gy was associated with a trend to improved local control [13]. However, advanced radiation techniques such as proton therapy (70-77.4 GyRBE) have demonstrated modest tumor control rates in retrospective and prospective series in the post-operative setting [14,15] as well as the pre-operative setting [13,16] with tumor control rates above 80% at 3-5 years. Carbon ions share the physical advantages of proton therapy and several biological advantages as well, including the ability to break clustered DNA breaks and more efficient cell killing, even in hypoxic regions [13]. The outcomes of patients treated with carbon ions have demonstrated modest outcomes [17], and even comparative series [18] demonstrate that either of these approaches should be considered for patients with spine chordomas.

One challenge to treating chordoma patients in the post-operative setting is the presence of metallic surgical hardware which creates challenges for visualizing any residual or recurrent disease, obscuring delineation of nearby CNS OARs, and reducing dosimetric coverage of the target areas. In an experience of 100 patients with spine chordomas, 39% of whom had prior surgical hardware placed (primarily titanium) and were treated with adjuvant proton therapy to 74 Gy, surgical stabilization was independently associated with worse disease control [19]. Consensus recommendations from the Particle Therapy Cooperative Group recommend the placement of hardware with cross-links above and below the intended target volume, the use of alternate materials with reduced artifacts, and treatment planning techniques, to maximize the visualization and to minimize the negative impacts of hardware placement in patients treated with particle therapies [20]. Ultimately, sometimes multi-modality planning is required to overcome these challenges. In this case, a PEEK synthetic plastic interbody cage was utilized over more common titanium interbody implants in order to mitigate and minimize radiographic artifact for radiation planning.

## Conclusions

Surgical management of cervical spine chordoma represents a challenging and overall rare clinical entity. Successful treatment requires not only complete surgical resection but also the anticipation of the potential risks of the surgical procedure. In this case, an unintended, although anticipated, complication in the form of left VA injury was managed with intra-operative sacrifice and a more complex surgical reconstruction. This technique resulted in the adequate reconstruction and stabilization of the vertebral elements, while maintaining adequate protection of the injured blood vessel, and adequate post-operative visualization of the anatomy, with ultimately a positive clinical result for the patient.

## References

[1] McMaster ML, Goldstein AM, Bromley CM, Ishibe N, Parry DM (2001). Chordoma: incidence and survival patterns in the United States, 1973-1995. Cancer Causes Control.

[2] Bergh P, Kindblom LG, Gunterberg B, Remotti F, Ryd W, Meis-Kindblom JM (2000). Prognostic factors in chordoma of the sacrum and mobile spine: a study of 39 patients. Cancer.

[3] Barrenechea IJ, Perin NI, Triana A, Lesser J, Costantino P, Sen C (2007). Surgical management of chordomas of the cervical spine. J Neurosurg Spine.

[4] Park H, Choi Y, Lee S (2024). The clinical outcomes of cervical spine chordoma: a nationwide multicenter retrospective study. Neurospine.

[5] Sciubba DM, Schwab JH (2021). Chordoma of the Spine: A Comprehensive Review. Chordoma of the Spine. Springer.

[6] Sakellariou E, Benetos IS, Evangelopoulos DS (2023). Incidence of vertebral artery injury in patients undergoing cervical spine trauma surgery in correlation with surgical approach: a review. Medicine (Baltimore).

[7] Neo M, Fujibayashi S, Miyata M, Takemoto M, Nakamura T (2008). Vertebral artery injury during cervical spine surgery: a survey of more than 5600 operations. Spine (Phila Pa 1976).

[8] Yi HJ (2022). Epidemiology and management of iatrogenic vertebral artery injury associated with cervical spine surgery. Korean J Neurotrauma.

[9] Wu EM, Khan NR, Yunga Tigre J, Abdelsalam A, Cote I, Ivan ME, Morcos JJ (2024). Vertebral artery pseudoaneurysm after extreme lateral transcondylar transodontoid approach for clival chordoma: 2-dimensional operative video. Oper Neurosurg.

[10] Sun Y, Shi YM, Xu P (2022). The clinical research progress of vertebral artery dominance and posterior circulation ischemic stroke. Cerebrovasc Dis.

[11] Hsieh PC, Gallia GL, Sciubba DM (2011). En bloc excisions of chordomas in the cervical spine: review of five consecutive cases with more than 4-year follow-up. Spine (Phila Pa 1976).

[12] Yolcu Y, Wahood W, Alvi MA, Kerezoudis P, Okuno SH, Foote RL, Bydon M (2019). Evaluating the role of adjuvant radiotherapy in the management of sacral and vertebral chordoma: results from a national database. World Neurosurg.

[13] Konieczkowski DJ, DeLaney TF, Yamada YJ (2020). Radiation strategies for spine chordoma: proton beam, carbon ions, and stereotactic body radiation therapy. Neurosurg Clin N Am.

[14] Indelicato DJ, Rotondo RL, Begosh-Mayne D, Scarborough MT, Gibbs CP, Morris CG, Mendenhall WM (2016). A prospective outcomes study of proton therapy for chordomas and chondrosarcomas of the spine. Int J Radiat Oncol Biol Phys.

[15] Baumann BC, Lustig RA, Mazzoni S (2019). A prospective clinical trial of proton therapy for chordoma and chondrosarcoma: feasibility assessment. J Surg Oncol.

[16] DeLaney TF, Liebsch NJ, Pedlow FX (2009). Phase II study of high-dose photon/proton radiotherapy in the management of spine sarcomas. Int J Radiat Oncol Biol Phys.

[17] Imai R, Kamada T, Araki N (2016). Carbon ion radiation therapy for unresectable sacral chordoma: an analysis of 188 cases. Int J Radiat Oncol Biol Phys.

[18] Iannalfi A, D'Ippolito E, Riva G (2020). Proton and carbon ion radiotherapy in skull base chordomas: a prospective study based on a dual particle and a patient-customized treatment strategy. Neuro Oncol.

[19] Snider JW, Schneider RA, Poelma-Tap D (2018). Long-term outcomes and prognostic factors after pencil-beam scanning proton radiation therapy for spinal chordomas: a large, single-institution cohort. Int J Radiat Oncol Biol Phys.

[20] Chhabra AM, Snider JW, Kole AJ (2024). Proton therapy for spinal tumors: a consensus statement from the Particle Therapy Cooperative Group. Int J Radiat Oncol Biol Phys.

